# Leveraging Bulk and Single-Cell RNA Sequencing Data of NSCLC Tumor Microenvironment and Therapeutic Potential of NLOC-15A, A Novel Multi-Target Small Molecule

**DOI:** 10.3389/fimmu.2022.872470

**Published:** 2022-05-17

**Authors:** Bashir Lawal, Alexander T. H. Wu, Hsu-Shan Huang

**Affiliations:** ^1^ Ph.D. Program for Cancer Biology and Drug Discovery, College of Medical Science and Technology, Taipei Medical University and Academia Sinica, Taipei, Taiwan; ^2^ Graduate Institute for Cancer Biology and Drug Discovery, College of Medical Science and Technology, Taipei Medical University, Taipei, Taiwan; ^3^ TMU Research Center of Cancer Translational Medicine, Taipei Medical University, Taipei, Taiwan; ^4^ The PhD Program of Translational Medicine, College of Medical Science and Technology, Taipei Medical University, Taipei, Taiwan; ^5^ Clinical Research Center, Taipei Medical University Hospital, Taipei Medical University, Taipei, Taiwan; ^6^ Graduate Institute of Medical Sciences, National Defense Medical Center, Taipei, Taiwan; ^7^ School of Pharmacy, National Defense Medical Center, Taipei, Taiwan; ^8^ PhD Program in Biotechnology Research and Development, College of Pharmacy, Taipei Medical University, Taipei, Taiwan

**Keywords:** NLOC-15A, multitarget small molecule, non-small-cell lung cancer (NSCLC), epidermal growth factor receptor (EGFR), hippo pathway

## Abstract

Lung cancer poses a serious threat to human health and has recently been tagged the most common malignant disease with the highest incidence and mortality rate. Although epidermal growth factor (EGFR)-tyrosine kinase inhibitors (TKIs) have significantly improved the prognosis of advanced non-small cell lung cancer (NSCLC) patients with EGFR mutations, patients often develop resistance to these drugs. There is therefore a need to identify new drug candidates with multitarget potential for treating NSCLC. We hereby provide preclinical evidence of the therapeutic efficacy of NLOC-015A a multitarget small-molecule inhibitor of EGFR/mitogen-activated protein (MAP) kinase kinase 1 (MAP2K1)/mammalian target of rapamycin (mTOR)/yes-associated protein 1 (YAP1) for the treatment NSCLC. Our multi-omics analysis of clinical data from cohorts of NSCLC revealed that dysregulation of EGFR/MAP2K1/mTOR/YAP1 signaling pathways was associated with the progression, therapeutic resistance, immune-invasive phenotypes, and worse prognoses of NSCLC patients. Analysis of single-cell RNA sequencing datasets revealed that MAP2K1, mTOR, YAP1 and EGFR were predominantly located on monocytes/macrophages, Treg and exhaustive CD8 T cell, and are involved in M2 polarization within the TME of patients with primary and metastatic NSCLC which further implied gene’s role in remodeling the tumor immune microenvironment. A molecular-docking analysis revealed that NLOC-015A bound to YAP1, EGFR, MAP kinase/extracellular signal-related kinase kinase 1 (MEK1), and mTOR with strong binding efficacies ranging –8.4 to –9.50 kcal/mol. Interestingly, compared to osimertinib, NLOC-015 bound with higher efficacy to the tyrosine kinase (TK) domains of both T790M and T790M/C797S mutant-bearing EGFR. Our *in vitro* studies and sequencing analysis revealed that NLOC-015A inhibited the proliferation and oncogenic phenotypes of NSCLC cell lines with concomitant downregulation of expression levels of mTOR, EGFR, YAP1, and MEK1 signaling network. We, therefore, suggest that NLOC-015A might represent a new candidate for treating NSCLC *via* acting as a multitarget inhibitor of EGFR, mTOR/NF-κB, YAP1, MEK1 in NSCLC.

## Introduction

Lung cancer poses a serious threat to human health and was recently tagged the second most common malignant disease with the highest incidence and mortality rate in Asia and the world at large (1, 2). With an estimated 1.8 million deaths, lung cancer is the leading cause of cancer-associated deaths globally (1). Non-small-cell lung cancer (NSCLC) and small-cell lung cancer (SCLC) are the most common classifications for diagnosing lung cancer. However, NSCLC comprises about 80%~85% of all lung cancers (3) including adenocarcinomas and squamous cell carcinomas (4) with a 5-year survival rate of <15% (5), and thus it has become the focus of studying lung cancer resistance and drug target mechanisms.

Epidermal growth factor (EGF) receptor (EGFR) signaling plays vital roles in angiogenesis, cell proliferation, apoptosis inhibition, metastasis, and drug sensitivity and resistance (6). Receptor tyrosine kinases (RTKs) are high-affinity cell surface receptors that play important roles in modulating growth factor signaling. EGFR is a four-members family of receptors: EGFR (or erbB1), HER2/neu (erbB2), HER3 (erbB3), and HER4 (erbB4) (7, 8). EGFRs bind ligands on their extracellular ligand-binding domains, and consequently activate their intracellular tyrosine kinase (TK) domains (8, 9) under normal physiological conditions. This process activates signaling pathways, such as the Ras/mitogen-activated protein kinase (MAPK), Janus kinase 2 (JAK2)/signal transducer and activator of transcription 3 (STAT3), and phosphatidylinositol 3-kinase (PI3K)/AKT, and stimulates downstream mechanisms involved in cell-cycle progression, motility, and cell proliferation and survival (10).

*EGFR* gene mutations frequently occur in exons 18~21 around the adenosine triphosphate (ATP)-binding pocket of the EGFR TK domain, while exon 19 deletions (35%~69%) and exon 21 L858R point mutations (21%~48%) occur following treatment with EGFR-tyrosine kinase inhibitors (TKIs) (11–13). These mutations render the receptor constitutively activated, independent of an extracellular ligand-binding event (14). Consequently, sustained hyperactivated downstream signaling pathways drive tumorigenesis and result in the emergence of NSCLC (15). About 50%~60% of Asian patients with NSCLC harbor *EGFR* mutations (16), and they are more common in females and non-smokers (11). NSCLC patients with these mutations often show improved prognoses and excellent responses to approved first- and second-generation EGFR-TKIs (17, 18). However, the development of EGFR T790M mutation-induced acquired drug resistance limits progression-free survival to a maximum of a year (19). Higher YAP expression was implicated in resistance to a number of cancer drugs (20, 21) including the first- and second-generation EGFR-TKIs (22), and treatment of lung cancer with growth factor (vascular endothelial growth factor (VEGF) and YAP) inhibitors provided promising results in previous studies (3, 23). However, to the best of our knowledge, no previous studies identified a molecule that can connectively modulate the Hippo, EGFR, RAS-MAPK, and PI3K-TOR pathways in NSCLC.

Tumor immune microenvironment (TIME) comprises extracellular components including cytokines, extracellular matrix, growth factors, hormones, etc.; several types of cells including stromal cells, immune cells, endothelial cells; fibroblasts, etc.) and cancerous cells (24). The complex interplay of the TIME components promotes immune escape, allowing for tumor progression and metastasis (25, 26). Accumulating evidence has suggested that several oncogenic molecules promote tumor immune evasion by regulating the infiltration of immune and immunosuppressive cells within the TME promoting and hence serving as important therapeutic targets (26–28). The extent of the heterogeneity of the TIME in primary and metastatic NSCLC and the role of mTOR/EGFR/YAP1/MAP2K1 in modulating the interplay of the TIME remains poorly characterized.

In this study, we employ multi-omics approaches based on bulk and single-cell transcriptomic datasets to investigate the role of MAP2K1, mTOR, YAP1 and EGFR in the tumor immune context of tumor microenvironment. Our omics analysis of clinical data from cohorts of NSCLC revealed that dysregulation of EGFR/MAP2K1/MTOR/YAP1 signaling pathways are associated with disease progression, anticancer drug resistance, tumor immune infiltration, immune-invasive phenotypes and worse prognosis of the cohorts. Further analysis using the single-cell RNA sequencing data demonstrated that MAP2K1, mTOR, YAP1 and EGFR were predominantly located on monocytes/macrophages, Treg and exhaustive CD8 T cell, and are involved in M2 polarization within the TME of patients with primary and metastatic NSCLC which further implied gene’s role in remodeling the tumor immune microenvironment. Interestingly, we demonstrated that NLOC-015A, a novel multitarget small molecule suppresses the proliferation and oncogenic phenotypes of NSCLC *via* inhibiting the EGFR, mTOR, MEK, and YAP1 signaling network. We, therefore, suggest that NLOC-015A might represent a new candidate for treating NSCLC. Our study has expanded our understanding of the complex regulatory mechanism of interaction and the role of EGFR/MAP2K1/MTOR/YAP1 within the TME of NSCLC and suggests the translational immunotherapeutic potential of NLOC-015A.

## Materials and Methods

### Differential Expression and Lung Cancer Context-Specific Network Interactions Analysis

We used TNMplot (29), a web tool for the differentially expressed gene (DEG) analysis in tumor, normal, and metastatic tissues to compare gene expression levels of EGFR, MAP2K1, mTOR, TEA domain family member 1 (TEAD1), and YAP1 between primary and metastatic lung cancer tissues. We analyzed lung cancer context-specific network interactions of hub genes using the Protein Interaction Network Analysis (PINA) platform (https://omics.bjcancer.org/pina/home). An interaction network was constructed based on the cutoff point of a tumor-type specific score of 2 and Spearman correlation of >0.28. All p-values were adjusted by false detection rate (FDR).

### Differential Phosphorylation Analysis

We explored protein phosphorylation data from the Clinical Proteomic Tumor Analysis Consortium (CPTAC) Confirmatory/Discovery dataset for EGFR, MAP2K1, mTOR, TEAD1, and YAP1 at different tumor stages of lung cancer patients. The protein phosphorylation levels were presented as Z-values which represent standard deviations (SDs) from the median across samples for a given cancer type. Log2[spectral count ratio values]s from CPTAC were first normalized within each sample profile and then normalized across samples (30).

### Prognostic Analysis

SurveExpress is a biomarker validation program for cancer gene expression (http://bioinformatica) (31). To conduct a survival analysis, we downloaded EGFR, MAP2K1, mTOR, TEAD1, and YAP1 expression profiles, survival days, and clinical data of six cohorts consisting of 1053 lung cancer patients from the Lung Meta-base. The cohorts were divided into two groups, (high- and low-expressions groups) based on the median expression levels of each gene. Patients were also sorted by prognostic index and divided into “High Risk” and “Low Risk” groups, according to the Maximized Risk Groups. Survival levels of the cohorts were merged with expression levels of the genes and the hazard ratio (HR) of each gene was estimated by fitting CoxPH using the risk group as a covariate.

### Tumor Immune Infiltration Analysis

We evaluated the DEGs expression-induced regulation of the abundance of immune cell infiltration of NSCLC tumor. The infiltration levels of the cytotoxic lymphocytes (B cells, CD4 naïve, CD4 T, CD8 naïve, CD8 T, cytotoxic, effector_memory T cell, MAIT cells, NK cells, and γδ T cells), T helper cells (Th1, Th17, Th2, and Tfh), immunosuppressive cells (regulatory T cell (Treg), Tr1 and cancer-associated fibroblast (CAF)) and myeloid cells (Dendritic cells, monocyte, macrophage, M1 macrophage and M2_macrophages (tumor-associated macrophages)) in HNSCCs were downloaded *via* the tumor IMmune Estimation Resource (TIMER2.0) (http://timer.cistrome.org/) server (32) and the GSCALite (http://bioinfo.life.hust.edu.cn/web/GSCALite/) server (33). The mRNA expression levels of EGFR, MTOR, YAP1, MAP2K1 were corellated with the levels of the infiltrating immune cells. All correlation analysis was conducted using the purity-adjusted partial Spearman’s rho value and statistical significance (p <0.05).

### Leveraging Single-Cell RNA Sequencing (scRNA-Seq) Datasets for Tumor Immune Microenvironment of Primary and Metastatic Sites of NSCLC

To characterize the role of EGFR/MAP2K1/mTOR/YAP1 within the tumor microenvironment of NSCLC at single-cell resolution, we explore the single-cell sequencing datasets (E-MTAB-6149 and GSE143423) of NSCLC *via* the Tumor Immune Single-cell Hub (34). The E-MTAB-6149 database consists of scRNA-seq from 5 patients with primary NSCLC (35), while GSE143423 datasets consist of scRNA-seq from 3 patients with metastatic NSCLC. For each of the datasets, we employ a uniform analysis pipeline – MAESTRO v1.1.0 to perform quality control, clustering and cell-type annotation. Model-based analyses of transcriptome and regulome (MAESTRO), is a comprehensive open-source computational workflow for the integrative analyses of single-cell RNA-seq (scRNA-seq) data from multiple platforms (36).Two levels of cell-type annotation including the malignancy and major-lineage were curated. We also performed an analysis of scRNA-seq expression of the genes for each cell type. The gene expression in each single cell was quantified as log2(TPM/10+1). We calculated differentially expressed genes (DEGs) between the cells from samples with NSCLC and the cells from controls. Furthermore, the enrichment of EGFR/MAP2K1/mTOR/YAP1 gene set signature was evaluated by using the GSEA module of the TISH.

### Analysis of Gene Association With Drug Resistance

We explore the GSCALite (http://bioinfo.life.hust.edu.cn/web/GSCALite/) server identify the association between the expression levels of the gene signature and the efficacy of several anticancer drug (33). Spearman correlation was used to correlate the messenger (m)RNA expression levels of the gene signature and the 50% inhibitory concentration (IC50) of the small molecules against various cells in the Therapeutics Response Portal (CTRP) and Genomics of Drug Sensitivity (GDSC) databases.

### Cell Lines and Culture

NSCLC cell lines (H441 and H1975) obtained from American Type Culture Collection (ATCC. Manassas, VA., USA) were cultured/subcultured at 90%~95% confluence in Roswell Park Memorial Institute (RPMI) 1640 medium supplemented with 10% fetal bovine serum (FBS) (Gibco) and 1% penicillin/streptomycin (Invitrogen, Life Technologies, Carlsbad, CA, USA) under standard incubator condition (37°C in 5% humidified CO_2_).

### Chemical Synthesis of NSC828786 and NSC828788

The starting material of diflunisal (2’,4’-difluoro-4-hydroxy-[1,1’-biphenyl]-3-carboxylic acid) was synthesized *via* a previously described stepwise protocol (37). Four millimolar (0.98) of diflunisal was prepared in anhydrous tetrahydrofuran (30 mL), mixed with 1 mL of thionyl chloride (14 mmol), and refluxed under a nitrogen atmosphere for 8 h. The resulting mixture was cooled to 27°C, steamed, and reacted with an anhydrous tetrahydrofuran solution of 3,4-difluoroaniline (0.4 mL, 4 mmol) for 14 h. Subsequently the reaction mixture was washed with ethyl acetate/n-hexane and extracted with ethyl acetate followed by stepwise washing with 10% NaHCO_3_ (15 mL), double-distilled (dd)H_2_O, and brine (10 mL), and dried over anhydrous MgSO_4_ to obtain the white compound, NSC828786 (N-(3,4-difluorophenyl)-2’,4’-difluoro-4-hydroxy-[1,1’-biphenyl]-3-carboxamide) (37). This was further cyclized to NSC828788 (6-(2,4-difluorophenyl)-3-(3,4-difluorophenyl)-2H-benzo[e] (1, 3) oxazine-2,4(3H)-dione) in the presence of methyl chloroformate and pyridine (38). The nuclear magnetic resonance (NMR) characterization of the compounds was deposited (37, 38).

### *In Silico* Evaluation of the Drug Likeness, Pharmacokinetics (PKs), and Physicochemical Properties of NSC828786 and NSC828788

The physicochemical properties, drug likeness, PKs, and medicinal chemistry of NSC828786 and NSC828788 were analyzed using SwissADME software (http://www.swissadme.ch) (39), and computer-aided Prediction of Biological Activity Spectra (PASS) web resources (http://way2drug.com/dr) (40). We used the blood-brain barrier (BBB) Prediction Server (https://www.cbligand.org/BBB/) which operates based on support vector machine (SVM) and LiCABEDS algorithms on four types of fingerprints of 1593 reported compounds (41) to analyze their BBB-permeation abilities. In addition, we also used the brain or intestinal estimated permeation method (BOILED-Egg) model (42) to further analyze the brain- and intestinal-permeation abilities of the compounds based on their lipophilicity and polarity. SMILES structures of the compounds were also uploaded to the SwissADME server and analyzed for the presence of pan-assay interference compound (PAIN) substructures (43).

### Molecular Docking

The NL0C-015A drug molecule was drawn in sybyl mol2 format using the Avogadro molecular builder and visualization tool vers. 1.XX (http://avogadro.cc/) (44). The mol2 format was further converted to protein data bank (PDB) format using PyMOL Molecular Graphics System, vers. 1.2r3pre from Schrödinger (https://pymol.org/edu/?q=educational/). The three-dimensional structure of the MEK1 FHA domain (PDB code: 5YYX), human mTOR complex 1~5.9 Å (PDB code: 5FLC), EGFR kinase domain T790M mutation (PDB code: 2JIT), and the human YAP and TEAD complex (PDB code: 3KYS) were retrieved from the Protein Data Bank (https://www.rcsb.org/). NL0C-015A and target molecules were converted into Auto Dock Pdbqt format using AutoDock Vina (45). All water molecules were removed, and hydrogen atoms and Kolmman charges were added to the proteins. The docking results were visualized using PyMOL software, and the binding pocket of the proteins and Pymol interactions of NL0C-015A in the binding pocket of target active sites were identified using the Discovery Studio Visualizer (46) and (CASTp) program (http://cast.engr.uic.edu) (47). Hydrophobic contacts in the ligand-receptor complexes were visualized with the aid of the protein-ligand interaction profiler server (https://plip-tool.biotec.tu-dresden.de/plip-web/plip/index) (48).

### Cell-Viability (Sulforhodamine B) Assay

A cell-viability assay was conducted using the sulforhodamine B (SRB; Sigma-Aldrich, Taipei, Taiwan) reagent as described previously (49). Cells were harvested at 80% confluence and seeded in 96-well plates (10^4^ cells/well) for 24 h followed by drug treatment. After 48 h of drug treatment, cells were fixed with trichloroacetic acid (TCA; 10%) for 1 h and then stained with 0.4% (w/v) SRB. Unbound SRB was washed out with 1% (v/v) acetic acid and allowed to dry overnight. The contents of the plate were further solubilized in 20 mM Tris buffer, and the absorbance was recorded at 562 nm with the aid of a microplate reader (Molecular Devices, Sunnyvale, CA, USA).

### *In Vitro* Anti-Cancer Analysis of NLOC-015A

The NL0C-015A was evaluated for *in vitro* activities against the nine cell lines subset of human lung cancers including A549/ATCC, EKVX, HOP-62, HOP-92, NCI-H226, NCI-H23, NCI-H322M, NCI-H460 and NCI-H522 using the SRB protocol (49). The compound was screened for it anti-cancer activities at five different concentrations of 0, 0.1, 1.0, 10, and 100 μM. The activity of the drug on each cell line was calculated and expressed as the drug concentration causing a 50% reduction in the net protein increase in control cells during the drug incubation (GI_50_), and drug concentration causing total growth inhibition (TGI) (28):


$${\text{GI}}_{50}\left(\text{μM}\right):\;\left[\left(\text{Ti}-\text{Tz}\right)/\left(\text{C}-\text{Tz}\right)\right]\times \;100=\;50,$$

TGI (μM): Ti = Tz.

where Tz is the absorbance at time 0, C is the absorbance of the control after 48 h without treatment and Ti is the absorbance of drug-treated cells after 48 h. When the maximum dose tested (100 μM) did not meet the required effect on the cell line, the GI_50_ and TGI were expressed as greater than the maximum drug concentration tested (50).

### Colony-Formation Assay

A method modified from Franken et al. (51) was used to assess the effect of NL0C-015A treatments on colony formation by H441 and H1975 cells. Briefly, 500 cells were seeded in six-well plates and treated with NL0C-015A. Cells were allowed to grow for 5 days, and the colony-formation inhibitory effect of the drug was assessed relative to untreated cells using an SRB fixation protocol.

### Cell-Migration Assay

A 10^4^ cells/well of NSCLC cells (H441 and H1975) were seeded in the serum-free medium into the upper chamber of the 24-transwell chambers (pore size of 8 μm; ThermoFisher, Taipei, Taiwan), while 20% FBS containing media was added to the lower chamber and incubated for 24 h at 37°C (52). After incubation, the upper chamber was carefully cleared of the non-invaded cells while the invaded cells in the lower chamber side were subjected to SRB staining, and photographed under a microscope.

### Tumor Sphere Formation Assay

The sphere formation assay was conducted according to the method described by Ma et al. (53). Briefly, the NSCLC cells (H441 and H1975) were seeded into the ultra-low-attachment six-well plates (10^5^ cells per well) containing stem cell medium; serum-free RPMI 1640 medium supplemented with B27 and 20 ng/mL human basic fibroblast growth factor (bFGF) (Invitrogen, Grand Island, NY, USA), and epidermal growth factor (20 ng/mL, Millipore, Bedford, MA). The plates were incubated in the presence or absence of drug for 48 h. The aggregated spheres (diameter >50 µm) were counted and photographed with an inverted phase-contrast microscope.

### Western Blot Analysis

Total protein lysates from NL0C-015A-treated and untreated cells were harvested using a protein lysis buffer (containing proteinase inhibitors and phosphatase inhibitors, RIPA). Protein lysates (25 µg) were denatured and separated using a 10% sodium dodecylsulfate polyacrylamide gel electrophoresis (SDS-PAGE) gel (54). Proteins were transferred onto nitrocellulose membranes and blocked in 5% skim milk followed by washing with TBST (0.2% Tween-20, 50 mM Tris-HCl at pH 7.5, and 150 mM NaCl). Following overnight incubation (at -4°C) with respective primary antibodies. The membrane was incubated with appropriate horseradish peroxidase-conjugated secondary antibodies for 60 min. Protein-antibody interactions were detected with an enhanced chemiluminescence (ECL) kit (ECL-Plus, Amersham Pharmacia Biotech, Piscataway, NJ, USA), and the band image was captured using Imaging System (Upland, CA, USA). β-actin was used as an internal control to confirm equal gel loading.

### RNA Sequencing Analysis

After 48 hr of drug treatment, the total RNA was extracted from the treated as well as the control cells using TRIzol reagent (Thermo Fisher Scientific), according to the manufacturer’s instructions, and were subjected to RNA sequencing analysis

### Statistical Analysis

Replicates of the experimental data were analyzed using GraphPad Prism vers. 6.04 for Windows (GraphPad Software, La Jolla, CA, USA). Results were computed as the mean ± SD of the replicates. Data from treatment groups were compared with the control using Student’s *t*-test. Data were considered statistically significance at *p*<0.05 (*), *p*<0.001 (**), and *p*<0.0001 (***).

## Results

### Dysregulation of the *EGFR/MAP2K1/mTOR/TEAD1/YAP1* Signaling Axis Is Associated With Progression and Worse Prognoses of NSCLC

**Figure 1:**
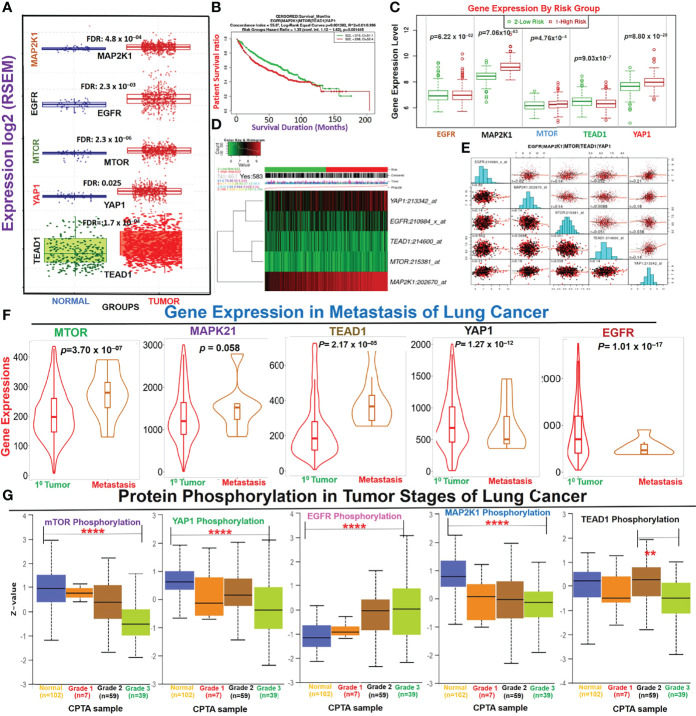
Dysregulation of the *EGFR/MAP2K1/mTOR/TEAD1/YAP1* signaling axis is associated with the progression and worse prognoses of non-small-cell lung cancer (NSCLC). **(A)** Boxplot of differential expressions of *EGFR, MAP2K1, mTOR, TEAD1*, and *YAP1* between lung cancer tumor tissues and adjacent normal tissues. **(B)** Kaplan-Meir plot of differential survival between differentially expressed genes. **(C)** Box plots of the differential gene expression values across gene groups together with the p-value of the corresponding difference. Box plots compare the difference of gene expression between risk groups using a t-test. High risk: red; low risk: green **(D)** Heat map representation of the survival risks of NSCLC cohorts. Patients were sorted by prognostic index and divided into “Low Risk” and “High Risk” groups, according to the “Maximized Risk Groups. Heat map shows the expression of each gene (rows) along samples (columns) in risk groups. Low expression is represented in green grades and high expression in red grades. **(E)** Gene expression correlation plot in lung cancer cohorts. The colors represent the class of the censored patients. Red = death and black = alive. **(F)** Violin plots of differential expression levels of the genes between primary and metastatic NSCLC. **(G)** Box-plot of differential phosphorylation statuses of the *EGFR, MAP2K1, mTOR, TEAD1,* and *YAP1* proteins in various NSCLC tumor grades **p < 0.01, ****p < 0.0001.

We analyzed differential expression patterns of *EGFR, MAP2K1, mTOR, TEAD1*, and *YAP1* between lung cancer tumor tissues and adjacent normal tissues and found that *EGFR* (*p*=2.3×10^–03^), *MAP2K1* (*p*=4.8×10^–04^), *mTOR* (*p*=2.3×10^–06^), *TEAD1* (*p*=1.7×10^–04^), and *YAP1* (*p*=0.025) were significantly overexpressed in lung cancer tumor tissues compared to adjacent normal tissues (**Figure 1A**). Interestingly, we found that cohorts with high expression levels of these DEGs exhibited higher risks (HR=1.35) and shorter survival durations (*p*=0.00145) than cohorts with low expression levels (**Figure 1B**). In addition, each of the genes demonstrated independent associations with the high risk and poor survival of the cohorts: *EGFR* (*p*=6.22×10^−02^), *LATS1* (*p*=5.26×10^−59^), *MAP2K1* (*p*=7.06×10^−63^), *mTOR* (4.76×10^−4^), *TEAD1* (9.03×10^−7^), and *YAP1* (8.80×10^−20^) (**Figures 1C, D**). We conducted a gene expression correlation analysis and found that the gene signature exhibited significant (*p*<0.05) gene expression correlations in lung cancer cohorts (**Figure 1E**). Furthermore, we found that expression levels of these genes were associated with tumor metastasis; *MAP2K1, mTOR*, and *TEAD1* were significantly overexpressed, while *YAP* and *EGFR* were under-expressed in metastasized lung cancer compared to primary tumors (**Figure 1F**). In addition, we queried the phosphorylation status of the proteins and found that mTOR and MAP2K1 showed consistent increases (*p*<0.05) in dephosphorylation, while EGFR was consistently hyper-phosphorylated (*p*<0.05) in higher tumor grades (normal, grades I~III). However, the p-YAP1 and p-TEAD showed a similar patterns of increased expressions from grade I-II and consistent decreases from grade II to III (**Figure 1G**).

### Genetic Alterations of *EGFR/MAP2K1/mTOR/TEAD1/YAP1* Mediate Other Oncogenic Interactions and Worse Prognoses of NSCLC Cohorts

**Figure 2:**
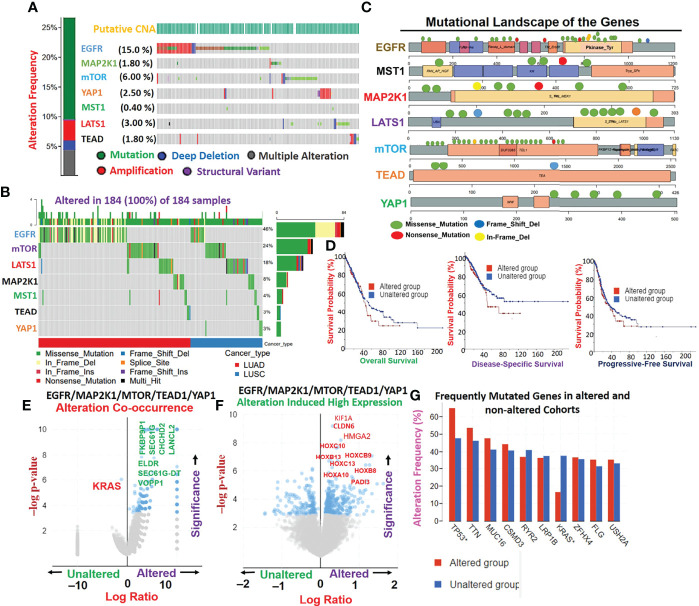
Genetic alterations *EGFR/MAP2K1/mTOR/TEAD1/YAP1* mediate other oncogenic interactions and produce worse prognoses of non-small-cell lung cancer (NSCLC) cohorts. **(A)** Genetic alteration plots, **(B)** waterfall plots of mutational frequencies, and **(C)** mutational landscape plots of *MST1/LATS1*/*EGFR/MAP2K1/mTOR/TEAD1*, and *YAP1* in TCGA cohorts of lung cancer. **(D)** Kaplan-Meir survival plots of differential survival of cohorts with genetic alterations of *EGFR, MAP2K1, mTOR, TEAD1*, and *YAP1*. **(E)** Gene alteration co-occurrence **(F)** and gene alteration-induced protein expression plots of *EGFR/MAP2K1/mTOR/TEAD1/YAP1* in lung cancer cohorts. **(G)** Bar plots of the most frequently mutated genes associated with *EGFR/MAP2K1/mTOR/TEAD1/YAP1*.

Genetic alterations of *EGFR, MAP2K1, mTOR, TEAD1*, and *YAP1* in The Cancer Genome Atlas (TCGA) cohorts of lung cancer occurred at respective frequencies of 15.0%, 1.80%, 6.00%, 1.80%, and 2.50% (**Figure 2A**). Mutations were the most prevalent genetic alterations occurring in all (100%) of the cohorts with genetic alterations (**Figure 2B**). Specifically, missense mutations and in-frame deletions were the most commonly occurring mutations (**Figures 2B, C**). By stratifying the mutation profile of individual cohorts, we found that the frequencies of protein changes in EGFR occurred at L858R (23; 27.0%), E746_A750del (16; 18.8%), E709_T710delinsD (3; 3.5%), L861Q (3; 3.5%), L747_E749del (3; 3.5%), S768I (2; 2.3%), T790M (2; 2.3%), L747_A750delinsP (2; 2.3%), L62R (2; 2.3%), and G719A (2; 2.3%), with other less-frequent mutations (**Figure 2C**). Protein changes in MAP2K1 included F53L, K57N, K57T, E102_I103del, C121S, G128V, R189*, M256T, and L313F (**Figure 2C**). Interestingly, we found that lung cancer cohorts with genetic alterations of *EGFR, MAP2K1, mTOR, TEAD1*, and *YAP1* exhibited worse prognoses of overall, disease-specific, and progression-free survival compared to patients with wild-type (WT) genes (**Figure 2D**). Genetic alterations of *EGFR/MAP2K1/mTOR/TEAD1/YAP1* co-occurred with genetic alterations of *ELDR, SEC61G-DT, VOPP1, FKBP9P1, SEC61G, CHCHD2*, and *LANCL2* (**Figure 2E**), and significantly (*p*<0.05) induced the overexpression of some onco-functional proteins including KIF1A, CLDN6, HMGA2, HOXC10, HOXB9, HOXB13, HOXC13, HOXB8, HOXA10, and PADI3 compared to their expression patterns in cohorts with WT *EGFR/MAP2K1/mTOR/TEAD1/YAP1* (**Figure 2F**). Furthermore, mutation of TTN, MUC16, CSMD3, RYR2, and LRP1B were found to be most frequently associated with EGFR/MAP2K1/mTOR/TEAD1/YAP1 alterations in NSCLC (**Figure 2G**).

### *EGFR/MAP2K1/mTOR/TEAD1/YAP1* Mediate Lung Cancer-Specific Activation of Onco-Functional Molecules and Therapeutic Responses

**Figure 3:**
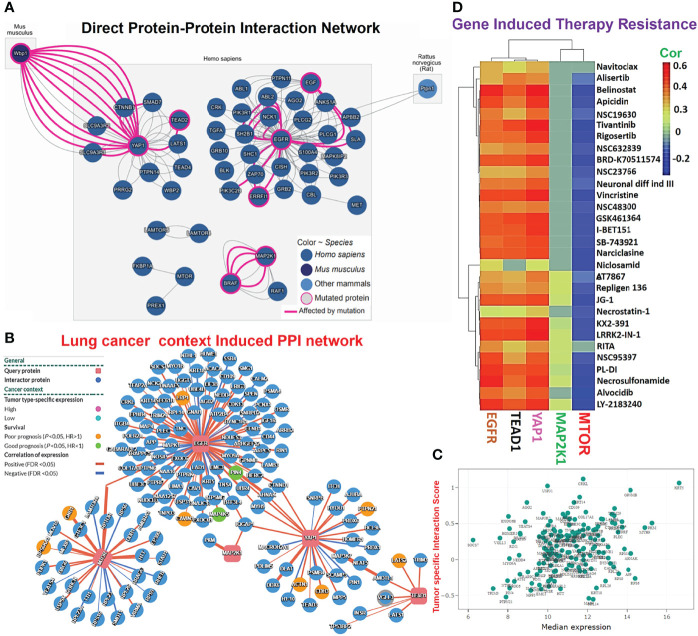
*EGFR/MAP2K1/mTOR/TEAD1/YAP1* mediate lung cancer-specific activation of onco-functional molecules and therapeutic responses. **(A)** Direct protein-protein interaction (PPI) network, **(B)** lung cancer-specific context-induced PP1 network, and **(C)** associated survival genes of *EGFR/MAP2K1/mTOR/TEAD1/YAP1* in lung cancer. **(D)** Heatmap of *EGFR/MAP2K1/mTOR/TEAD1/YAP1*-induced therapeutic resistance.

Our direct protein-protein interaction (PPI) network revealed that mutations of these genes significantly affected the function of a number of genes; *EGFR* affected *EGF*, *ANKS1A, APBB2, SLA, PLCG1, ABL2, PIK3R1, SH2B1*, *ZAP70, PIK3C2B*, and *ERRFI1*; YAP1 affected *TEAD2, SMAD7, CTNNB1, SLC9A3R2*, and *SLC9A3R1*, while MAP2K1 affected *BRAF* (**Figure 3A**). Our analysis of lung cancer context-specific interaction networks of hub genes revealed that deregulated expressions of *EGFR/MAP2K1/mTOR/TEAD1/YAP1* in lung cancer mediated abnormal expressions of 151 genes in total in 154 cancer-mediated interactions (**Figures 3B, C**). Furthermore, among these interactions, we identified *RPS16KA1, GNB1, LRP1, CAVIN1, ACTN1, CDH3, PTPN21*, and *LATS2* as important mediators of worse survival (*p*<0.05, HR>1) of the cohorts (**Figure 5B**). Enrichment analysis implicated several oncological pathways including wnt signaling, astrocyte differentiation, epithelial cell proliferation, cell morphogenesis, epidermis development, wound healing, cell proliferation, keratinocyte proliferation, hippo signaling, lung epithelium development, cellular senescence and EGFR TKI resistance in the pathological role of these gene in lung cancer (**Figure S1**). Interestingly, we found that the high expression levels of *EGFR, TEAD*, and *YAP1* were associated with resistance of cancer cell lines to several clinical anticancer drugs (navitoclax, alisertib, belinistat, tivantinib, niclosamide, necrostatin-1, alvocidib, etc.) and NSC drugs (NSC19630, NSC632839, NSC23766, NSC48300, and NSC95397). However, high expression of mTOR is favorable to the sensitivity of cancer cell lines to chemotherapy (**Figure 3D**).

### *EGFR/MAP2K1/mTOR/TEAD1/YAP1* Could Mediate Invasive Tumor Phenotypes and Worse Prognoses of NSCLC Cohorts *via* a Mechanism Involving Both T-Cell Exclusion and Dysfunctional Phenotypes

**Figure 4:**
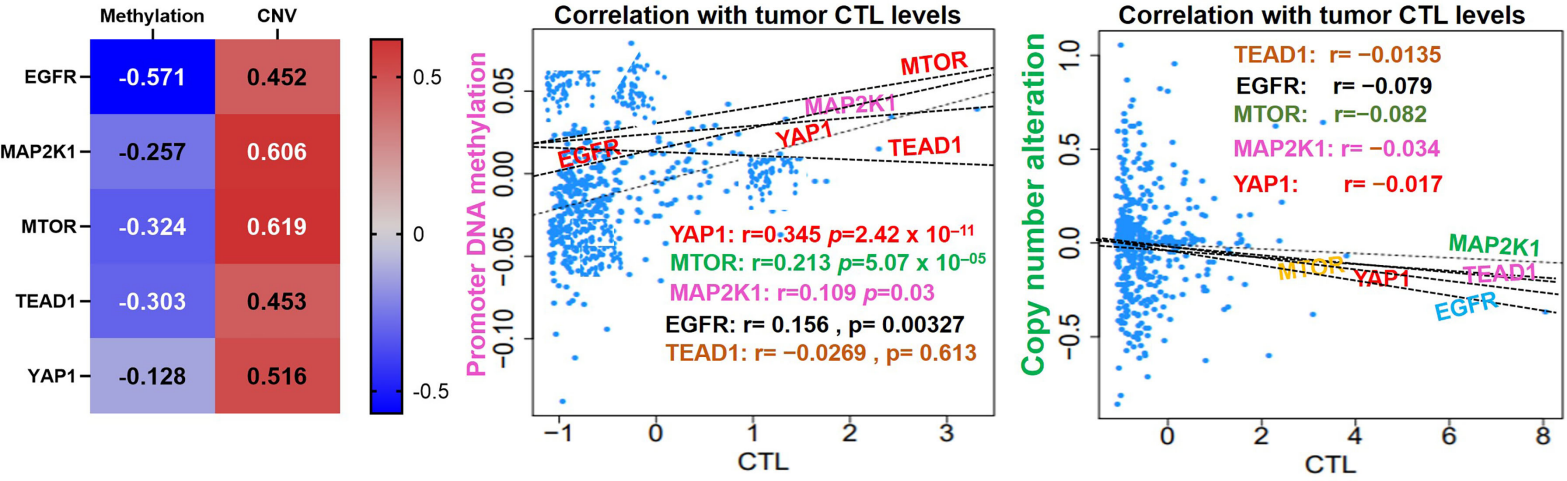
*EGFR/MAP2K1/MTOR/TEAD1/YAP1* is hypomethylated and exhibited high copy number alterations with a consequent negative impact on the levels of active CTL within the TME of NSCLC.

**Figure 5:**
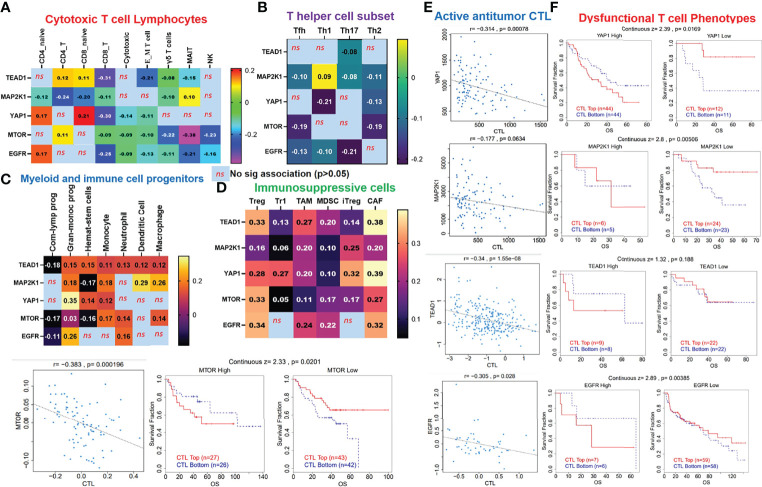
*EGFR/MAP2K1/mTOR/TEAD1/YAP1* could mediate invasive tumor phenotypes and worse prognoses of non-small-cell lung cancer (NSCLC) cohorts *via* mechanisms involving both T-cell exclusion and dysfunctional phenotypes. Heatmap plots of associations between mRNA expression levels of *EGFR/MAP2K1/mTOR/TEAD1/YAP1* and tumor infiltrations of **(A)** cytotoxic lymphocytes, **(B)** T helper cells, **(C)** myeloid cells, and **(D)** immunosuppressive cells in TCGA cohorts of NSCLC. **(E)** Scatterplot of correlations between mRNA expression levels of *EGFR/MAP2K1/mTOR/TEAD1/YAP1* and tumor levels of cytotoxic lymphocytes, and **(F)** Kaplan-Meier plot of dysfunctional T-cell phenotypes between cohorts with differential expression levels of *EGFR/MAP2K1/mTOR/TEAD1/YAP1* in NSCLC ns, non significant.

Our analysis of EGFR/MAP2K1/MTOR/TEAD1/YAP1 within the TME revealed that the gene signature is significantly hypomethylated and exhibited high copy number alterations with a consequent negative impact on the levels of active CTL within the TME of NSCLC (**Figure 4**). We found that messenger (m)RNA expression levels of the gene signature showed negative associations (≤-0, *p*<0.05) or no significant (*p*>0.05) associations with infiltration levels of cytotoxic lymphocytes (**Figures 5A, B**), cluster of differentiation 4 (CD4) naïve, CD4 T, CD8 naïve, CD8 T, cytotoxic, effector memory T, mucosal-associated invariant T (MAIT), natural killer (NK), and γδ T cells), T helper (Th) cells (Th1, Th17, Th2, and Tfh; **Figure 5B**), and common lymphoid progenitors (**Figure 5C**). Conversely, mRNA expression levels of the gene signature showed positive (*p*<0.05) correlations with infiltration levels of monocytes, granulocyte-monocyte progenitors (**Figure 5C**), and immunosuppressive cells (cancer-associated fibroblasts (CAFs), MDSCs, tumor-associated macrophages (TAMs), iTreg cells, and Tr1 cells) that are known mediators of T-cell exclusion (**Figure 5D**). Interestingly, we found that high expression levels of EGFR/MAP2K1/mTOR/TEAD1/YAP1 in lung cancer significantly decreased overall levels of active cytotoxic lymphocytes (**Figure 5E**), mediated dysfunctional T-cell phenotypes, and produced worse overall survival rates of lung cancer cohorts (**Figure 5F**). Collectively our results suggested that EGFR, MAP2K1, mTOR, TEAD1, and YAP1 could mediate invasive tumor phenotypes and produce worse prognoses *via* mechanisms involving both the T-cell exclusion and dysfunctional phenotypes.

### Single-Cell RNA Sequencing (scRNA-Seq) Datasets Revealed the Abundance and Immunosuppressive Role of *EGFR/MAP2K1/MTOR/TEAD1/YAP1* Within Tumor Microenvironment of Primary and Metastatic NSCLC

**Figure 6:**
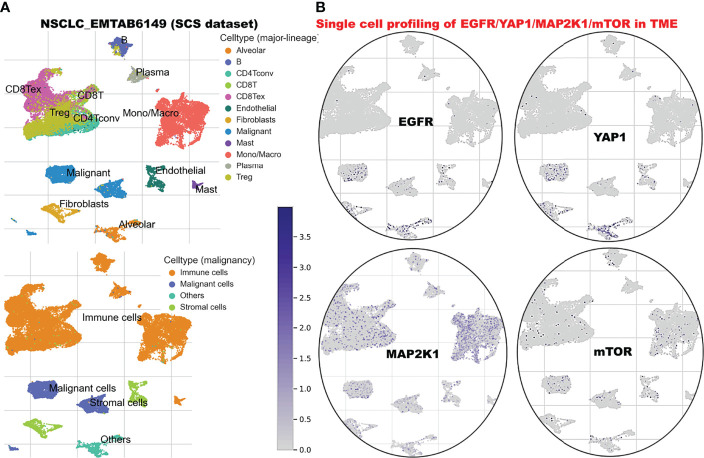
Single cell sequencing profiling of *EGFR/MAP2K1/MTOR/TEAD1/YAP1* in tumor microenvironment of primary NSCLC. **(A)** *t*-Distributed stochastic neighbor embedding plot of single-cell RNA-seq data from EMTAB6146. Identified clusters are represented by different colors (left). **(B)** The expression distribution of *EGFR/MAP2K1/MTOR/TEAD1/YAP1* in different cell types using single-cell resolution of EMTAB6146 dataset in TISCH database.

**Figure 7:**
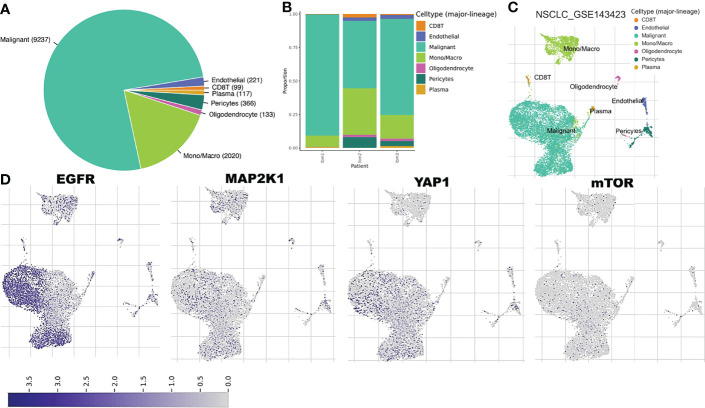
Single cell sequencing profiling of *EGFR/MAP2K1/MTOR/TEAD1/YAP1* in tumor microenvironment of metastatic NSCLC. **(A)** Cell type distribution and **(B)** bar plot patient distribution of cell-type major lineage of single cell sequencing data from metastatic NSCLC **(C)** *t*-Distributed stochastic neighbor embedding plot of single-cell RNA-seq data from GSE143423 datasets **(D)** The expression distribution of *EGFR/MAP2K1/MTOR/TEAD1/YAP1* in different cell types using single-cell resolution of GSE143423 dataset in TISCH database.

**Figure 8:**
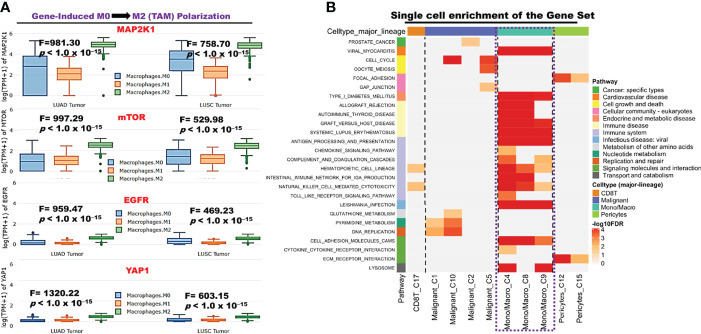
*EGFR/MAP2K1/MTOR/TEAD1/YAP1* induced macrophage polarization **(A)** Bar plot of expression levels of *EGFR/MAP2K1/MTOR/TEAD1/YAP1* at different macrophage state. **(B)** Enrichment Heatmap of the gene set expression in immune cell (Macrophage-monocyte) at single cell resolution.

We explore the single-cell RNA sequencing (scRNA-seq) datasets of NSCLC including the EMTAB6146 which consists of scRNA-seq from 5 patients with primary NSCLC, and the GSE143423 dataset which consists of scRNA-seq from 3 patients with metastatic NSCLC. The cell-type annotation of the patients with primary NSCLC at the levels of malignancy, and major-lineage, revealed the higher abundancy of the immune cells within the TME when compared with the malignant cells and stromal cells. Specifically, we found that the TME is mostly populated by monocytes and macrophages, regulatory T cells and Exhausted CD8 T Cells while a very low level of active CD8 T cells was found within the TME of patients with primary NSCLC (**Figure 6A**, **Table 1**). The differential expression analysis of scRNA-seq data revealed that the EGFR, mTOR, YAP1, and MAP2K1 are highly overexpressed in the malignant tissue. Meta-analysis of the differentially expressed genes within each cell of the TME, revealed higher expression levels of the genes particularly the MAP2K1 and mTOR occurs in the monocytes/macrophages, Treg and exhaustive CD8 T cell within the TME of patients with primary NSCLC (**Figures 6B** and **S2**). Analysis of scRNA-seq data of metastatic NSCLC in TME revealed a higher population of malignant cells when compared to other cells including the monocyte and macrophages (2020 single cells), endothelial (221 single cells), pericytes (366 single cells), oligodendrocytes (133 single cells), plasma (117 single cells) and a few CD8 T (99 single cells) were found (**Figures 7A–C**). When compared with the other genes, EGFR is the most highly differentially overexpressed gene in the malignant cells, monocytes and macrophages within the TME of metastatic NSCLC. MAP2K1 and YAP1 are also over-expressly populated in malignant cells, monocytes and macrophages within the TME of metastatic NSCLC (**Figure 7D**). Furthermore, we investigated the expression of EGFR/MTOR/YAP1/MAP2K1 in different states of macrophages, and the results demonstrated that genes’ expression were significantly higher in M2 macrophages compared to M0 and M1 macrophages (**Figure 8A**), suggesting that EGFR/MTOR/YAP1/MAP2K1 are involved in M2 polarization, an essential step for the remodeling of tumor immune microenvironment (TIME) (55). Furthermore, the high expression of these gene signature in macrophage achieved enrichment in several onco-immunological pathways including the complement and coagulation cascade, intestinal immune network, Toll like receptor signaling pathways and several infectious diseases (**Figure 8B**). Collectively, our analysis of single cell sequencing data suggested that the EGFR/MAP2K1/MTOR/TEAD1/YAP1 are highly expressed in the immune and malignant cells of TME with consequent influence on TAM-mediated immunosuppressive TME of primary and metastatic NSCLC.

**Table 1:** Differential expression of *EGFR/MAP2K1/mTOR/YAP1* in different cell cluster of NSCLC based on single-cell RNA sequencing data.

Cluster	Cell type	Major-lineage	Minor-lineage	Gene	Log2 FC	Percentage (%)	Adjusted p-value
3	Immune cells	Mono/Macro	M1	MTOR	0.34	60.3	1.52e-107
4	Immune cells	Mono/Macro	M2	MTOR	0.37	78.6	2.93e-265
9	Immune cells	Mono/Macro	Monocyte	MTOR	0.29	55.5	1.35e-53
17	Immune cells	Mono/Macro	Monocyte	MTOR	0.44	68.6	5.77e-69
20	Immune cells	Mono/Macro	M2	MTOR	0.28	88.6	9.11e-75
7	Malignant cells	Malignant	Malignant	MTOR	0.52	70	8.61e-231
15	Malignant cells	Malignant	Malignant	MTOR	0.41	70.1	1.06e-71
14	Stromal cells	Fibroblasts	Fibroblasts	MTOR	-0.29	33.7	5.64e-08
22	Stromal cells	Endothelial	Endothelial	MTOR	-0.47	14.2	9.18e-21
5	Immune cells	CD8T	CD8Tem	MTOR	-0.28	15.9	2.56e-77
2	Immune cells	CD4Tconv	Th17	MTOR	-0.34	18.4	3.62e-75
6	Immune cells	CD4Tconv	CD4Tn	MTOR	-0.46	11.8	3.69e-120
11	Immune cells	B	B	MTOR	-0.31	16.3	1.12e-54
4	Immune cells	Mono/Macro	M2	MAP2K1	0.31	49.3	0
9	Immune cells	Mono/Macro	Monocyte	MAP2K1	0.39	35.4	3.35e-89
20	Immune cells	Mono/Macro	M2	MAP2K1	0.39	74.2	4.14e-203
21	Immune cells	Mast	Mast	MAP2K1	-0.29	7.8	1.75e-08
15	Malignant cells	Malignant	Malignant	EGFR	0.33	27.8	0
23	Malignant cells	Malignant	Malignant	EGFR	0.29	25.6	1.33e-124
13	Others	Alveolar	Alveolar	EGFR	0.42	20	1.03e-285
21	Immune cells	Mast	Mast	YAP1	0.78	11.3	0
7	Malignant cells	Malignant	Malignant	YAP1	0.29	48.3	1.08e-188
24	Malignant cells	Malignant	Malignant	YAP1	0.26	51.3	2.44e-19
11	Immune cells	B	B	YAP1	-0.29	6.7	1.06e-45
15	Malignant cells	Malignant	Malignant	YAP1	0.35	30.6	0
24	Malignant cells	Malignant	Malignant	YAP1	0.39	28.8	1.22e-67
14	Stromal cells	Fibroblasts	Fibroblasts	YAP1	0.32	14.2	4.78e-78
13	Others	Alveolar	Alveolar	YAP1	0.55	29.7	0

### Rationale for Drug Design *via* Scaffold-Hopping of Bioactive Compounds and Physicochemical Properties of NSC828786 (NLOC-014A) and NSC828788 (NLOC-015A)

**Figure 9:**
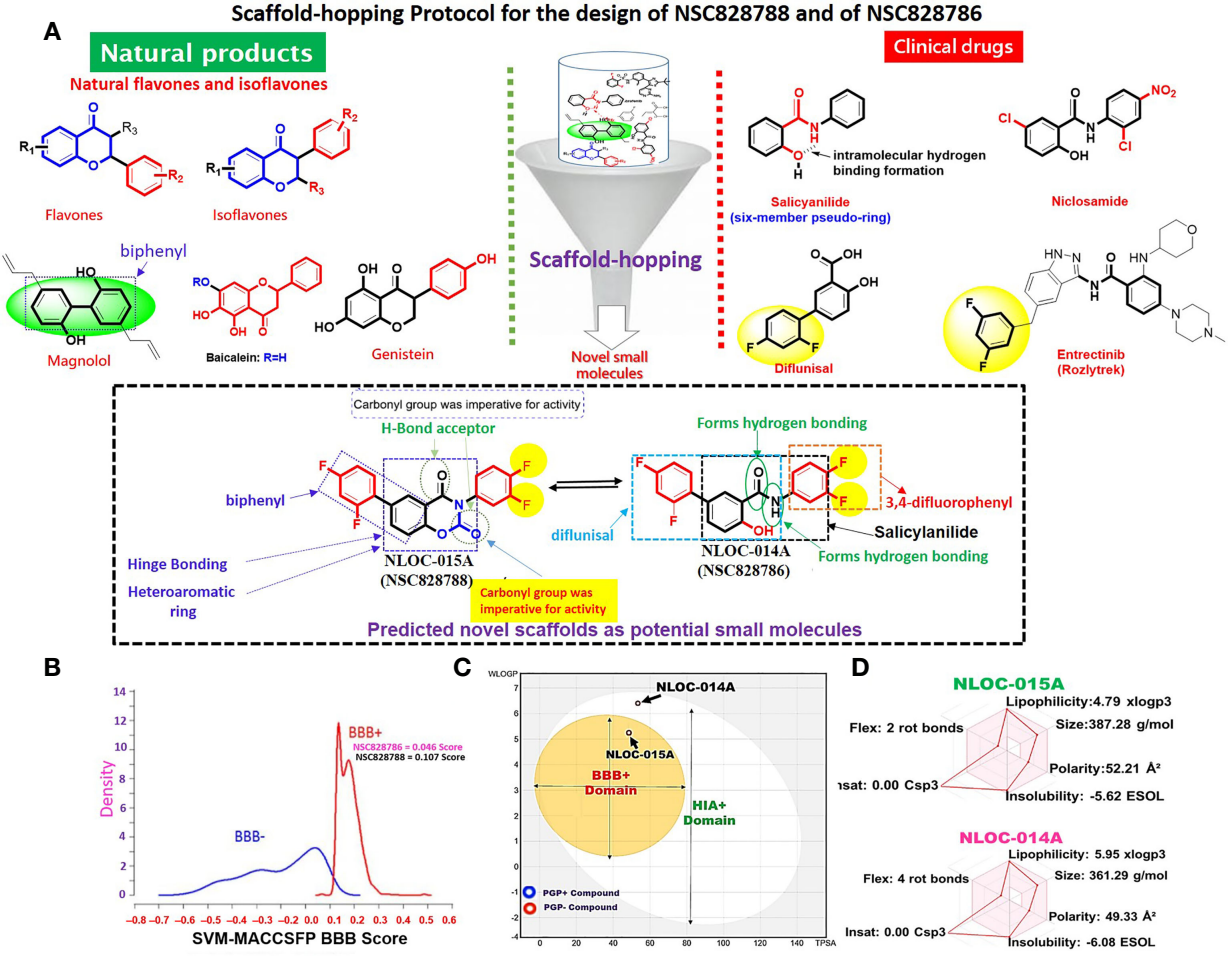
Rationale for drug design and physicochemical properties of NSC828786 (NLOC-014A) and NSC828788 (NLOC-015A). **(A)** Scaffold-hopping protocol for the design of NSC828788 and NSC828786. **(B)** Blood-brain barrier (BBB) plot of the support vector machine (SVM)/LiCABEDS algorithm and **(C)** BOILED-Egg model for BBB and human intestinal absorption (HIA) permeability of NSC828786 (NLOC-014A) and NSC828788 (NLOC-015A). **(D)** Bioavailability radar showing the physicochemical properties of NSC828786 (NLOC-014A) and NSC828788 (NLOC-015A).

Scaffold-hopping of bioactive compounds is an important approach for novel drug design and development (56). Biphenyl, flavones, and isoflavones are important natural product backbones, and several bioactive compounds containing these backbones were reported to have a vast range of biological activities, including antioxidative, anti-atherosclerosis, muscle relaxant, antimicrobial, anti-inflammatory, and anticancer effects (57, 58). A number of clinical drugs, e.g., diflunisal a salicylic acid derivative with several pharmacological activities (anticancer, anti-arthritis, analgesic, and anti-inflammatory properties) (59) and entrectinib (an anticancer drug), contain difluorophenyl as an important component responsible for their bioactivity. Niclosamide is a multipurpose compound with proven efficacy in treating several diseases, including oxidative stress, infections, metabolic disorders, inflammation, and cancers (60, 61). In the present study, scaffold-hopping of these natural bioactive compounds (flavones and isoflavones), biphenyl, difluorophenyl, and niclosamide led to the discovery of two structurally related novel small molecules: N-(3,4-difluorophenyl)-2’,4’-difluoro-4-hydroxy-[1,1’-biphenyl-3-carboxamide (NSC828786 or NLOC-014A) and 6-(2,4-difluorophenyl)-3-(3,4-difluorophenyl)-2H-benzo[e] [1,3]oxazine-2,4(3H)-dione (NSC828788 or NLOC-015A) (**Figure 9A**). Preclinical analysis of drug PKs plays an important role in the drug development process by providing a rationale for the selection of efficacious drug doses and treatment schedules (26). Herein, we evaluated the drug likeness and PK properties of these compounds. Our results also revealed good predictions for absorption, distribution, metabolism, excretion, toxicity (ADMET) properties, drug-likeness, adherence to Lipinski’s rules, and no pan-assay interference compounds (PAINS) alerts (**Figure 5D**). However, NSC828788 (NLOC-015A) demonstrated better PK properties and a higher BBB permeation score than NSC828786 (NLOC-014A) suggesting that NSC828788 would be a better drug candidate (**Figures 9B–D**).

### NSC828788 (NLOC-015A) Demonstrated Anticancer Activities and Potential for Targeting the *YAP1/EGFR/MEK1/mTOR* Signaling Network

**Figure 10:**
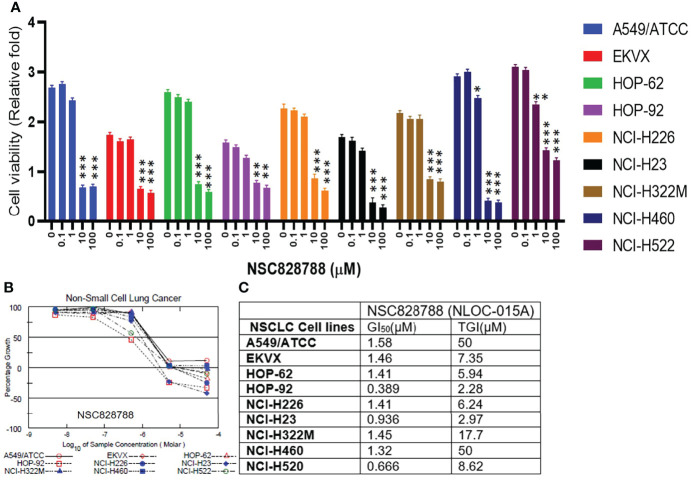
NSC828788 (NLOC-015A) demonstrated exhibited anticancer activities against NSCLC. **(A, B)**. Bar plot and dose-response curve of NSC828788 against panels of non-small-cell lung cancer (NSCLC). **(C)** IC50 and TG1 concentrations of NSC828788 (NLOC-015A) against panels of non-small-cell lung cancer. P-values were extrapolated as a function of cell viability of treatment with respect to control in the NCI-DTP screen, where *p < 0.05, **p < 0.001, and ***p < 0.001.

**Figure 11:**
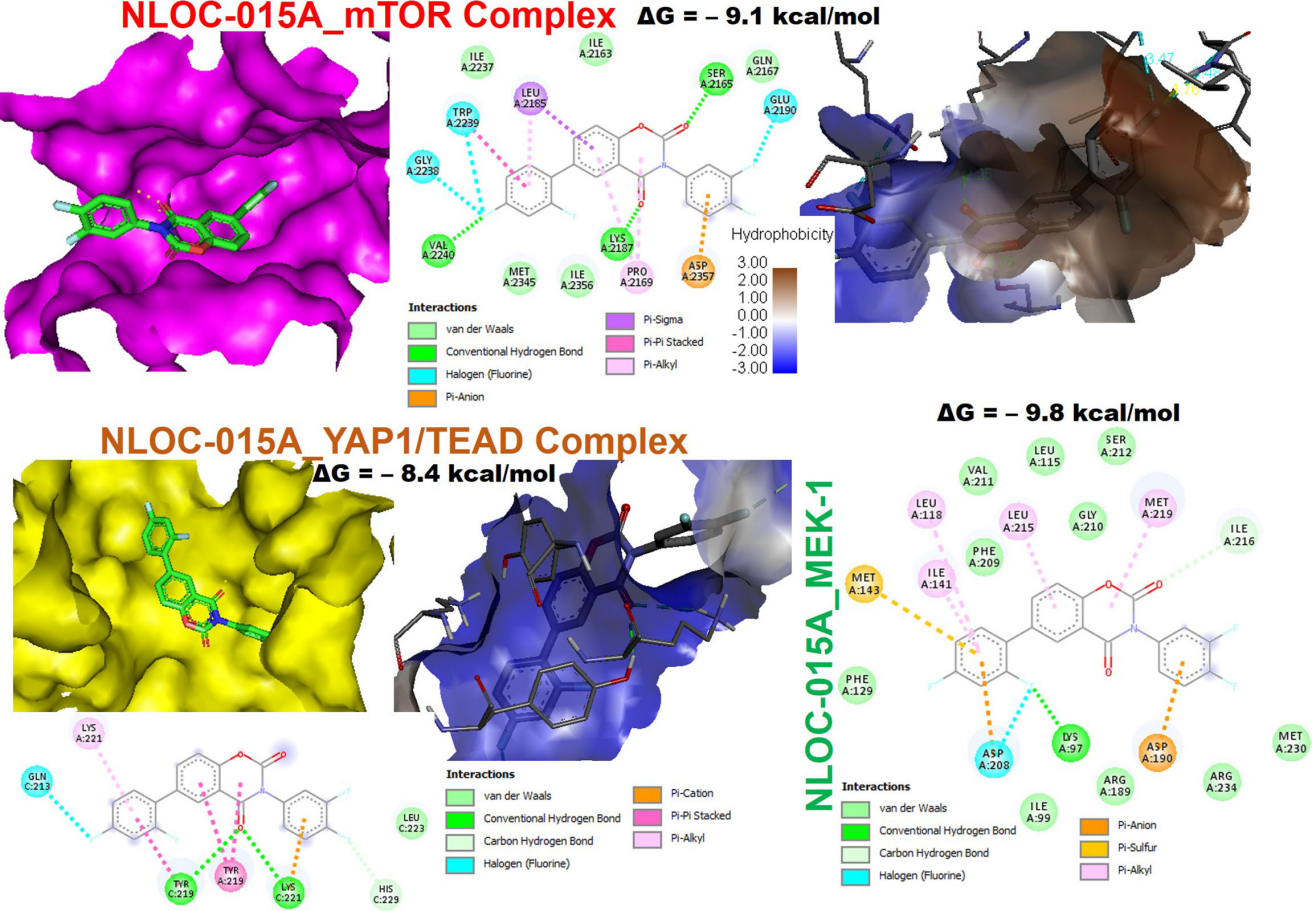
NSC828788 (NLOC-015A) has potential for targeting YAP1/EGFR/MEK1/mTOR signaling network. Two (2D) dimensional representation and the surface flip of the binding cavities of NSC828788 (NLOC-015A) interaction with mTOR, YAP1, and MEK1.

**Figure 12:**
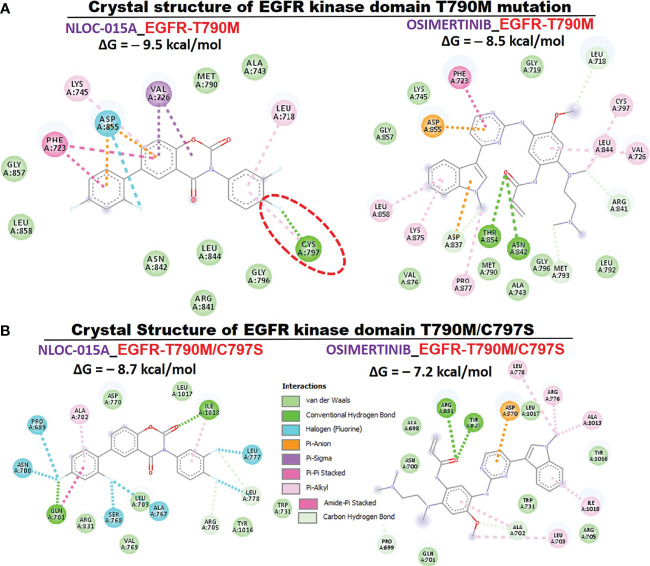
Two (2D) dimensional representation of NSC828788 (NLOC-015A) interaction with **(A)** T790M **(B)** T790M/C797S bearing mutant *EGFR*.

We first evaluated the *in vitro* anticancer activities of NSC828786 and NSC828788 against 60 human tumor cell lines panels of the National Cancer Institute (NCI). Interestingly, our results revealed that both NSC828786 and NSC828788 with single-dose (10 μM) treatment exhibited antiproliferative activities against all of the NCI-60 cell line panels of breast, prostate, renal, ovarian, colon, central nervous system (CNS), leukemia, and non-small cell lung cancers, and melanomas with NSC828788 demonstrating higher activities than NSC828786 (**Figure S3**). Similarly, we found that NSC828788 exhibited dose-dependent cytotoxic effects against nine panels of NSCLCs with IC_50_ values ranging 0.338~1.58 µM (**Figures 10A–C**). Subsequently, we explored the potential of NSC828788 as a therapeutic target against YAP1/EGFR/MEK1/mTOR through an *in silico* ligand-receptor interaction study. Interestingly, our results revealed that this compound (NLOC-015A) exhibited strong interactions and high affinities; –9.10, –9.50, –9.80, and –8.40 kcal/mol for the binding cavities of mTOR, EGFR, MEK-1, and YAP-TEAD, respectively (**Figure 11**). Our analysis of interactions between the NLOC-015A receptor complex revealed that NLOC-015A interacted with the targets through several conventional H-bonds, halogen bonds, and alkyl and multiple π-interactions (**Table 2**). Several van der Waals forces were found around the NLOC-015A backbone with the respective amino acid residues of target binding pockets. Furthermore, ligand-receptor complexes were stabilized by various hydrophobic contacts. Compared to osimertinib’s binding affinity, NLOC-015 bound with higher efficacy with T790M and T790M/C797S mutant-bearing EGFR (**Figure 12**). Altogether, the receptor-ligand interaction profile suggested the high potential of NLOC-015A to target the YAP1/EGFR/MEK1/mTOR signaling network.

**Table 2:** Docking profile of NSC828788 (NLOC-015A) with *YAP1/EGFR/MEK1/mTOR*.

	mTOR	EGFR	MEK-1	Yap1
ΔG (kcal/mol)	–9.10	–9.5	–9.80	–8.40
Conventional H-bonds	Ser2165 (1.76) Lys2187 (2.25) Val2240 (1.76)	Cys797 (2.69)	Ile216 (3.37) Lys97 (2.85) Asp208 (3.44)	Tyr219 (2.29) Lys221 (2.93) His229 (3.37)
Alkyl interactions	Pro2169, Leu2185	Leu718, Lys745	Met219, Leu215, Leu118 Ile141	
Fluorine	Glu2190 (2.25), Gly2238 (3.47), Trp2239 (3.48)	Asp855 (3.48)		Gln213 (3.28)
Pi-pi stacked	Trp223	Phe723		Thr219, Tyr219
PI-sigma	Leu2185	Val726		
Pi-anion	Asp2357	Asp855	Asp190, Asp208	
Pi-cation				Lys221
Pi-sulfur			Met143	
van der Waals forces	Ile2237, Ile2163, Gln2167, Met2345, Ile2356	Gly857, Leu858, Asn842, Arg841, Leu844, Gly796, Ala743, Met790	Val211, Leu115, Ser212, Phe209, Gly210, Phe129, Ile99, Arg189, Arg234, Met230	
Hydrophobic contacts	Leu718a (3.66)-atm5872 Phe723a (3.46)-atm5875 Lys745a (3.73)-atm5863	Leu718a (3.66)-atm5872 Phe723a (3.46)-atm5876 Lys745a (3.73)-atm5863	Ile141a (3.54)-atm2767 Asp190a (3.74)-atm2759	Ala208a (3.90)-atm1418 Phe224a (3.94)-atm1421 Leu279a (3.81)-atm1423 Thr309a (3.87)-atm1414 Val311a (3.87)-atm1411 Leu367a (3.98)-atm1425 Phe370a (3.80)-atm1425 Ile372a (3.95)-atm1415 Phe392a (3.76)-atm1419 Phe392a (3.91)-atm1425

mTOR, mammalian target of rapamycin; EGFR, epidermal growth factor receptor; MEK-1, mitogen-activated protein kinase/extracellular signal-regulated kinase kinase 1; Yap, yes-associated protein.

### NL0C-015A Exhibited Anti-NSCLC Activities *via* Modulation of Hippo- YAP1, *EGFR-MEK*, and NF-κB-TOR Pathways in NSCLC

**Figure 13:**
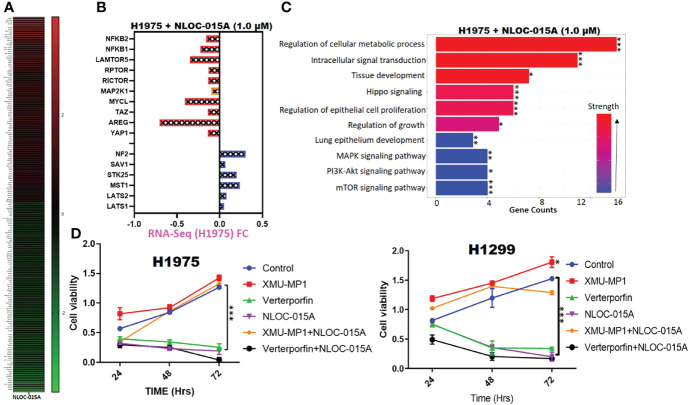
NL0C-015A exhibited anti-non-small-cell lung cancer (NSCLC) activities *via* modulation of Hippo, *EGFR-MEK*, and NF-κB-TOR pathways in NSCLC. **(A)** Heatmap of the top 100 upregulated and downregulated genes. **(B)** Plot of the effect of NLOC-015A treatment on expression of Hippo, *EGFR-MEK*, and NF-κB-TOR pathways. **(C)** GSEA plot of NLOC-015-mediated processes. **(D)** Cell viability plot showing that treatment with XMU-MP-1 attenuated the antiproliferative properties of NLO0C-015A in both H1975 and H1299 cells. *p < 0.05, **p < 0.01, ***p < 0.001.

To further explore the therapeutic mechanism of NLOC-015 in H1975 cells, we performed RNA-sequencing (RNA-Seq) to compare DEGs between control H1975 cells and NLOC-015-treated NSCLC cells. Our sequencing results revealed a total of 801 DEGs (*p*<0.05) with 348 downregulated and 453 upregulated genes. The top 100 upregulated and downregulated genes are displayed in a heatmap (**Figure 13A**). As expected, compared to control H1975 cells, NLOC-015-treated cells showed downregulation of negative effectors of the Hippo pathway, including YAP1, AREG, TAZ, MYCL, MAP2K1, RICTOR, RPTOR, and NF-κB and upregulation of positive effectors of the hippo pathway, including the LATS1/2, MST, STK25, SAV1, and NF2 (**Figure 13B**). Coherently, the gene set enrichment analysis (GSEA) of NLOC-015-mediated DEGs identified Hippo signaling, regulation of cellular metabolic processes, tissue development, intracellular signal transduction, MAPK, mTOR, and PI3K-AKT signaling, and lung epithelial development (**Figure 13C**). Furthermore, we investigated whether NL0C-015A exhibited antitumorigenic activities *via* modulation of the Hippo pathway in NSCLC. We cocultured H1299 and H1975 cells with XMU-MP1, an MST1/2 inhibitor, to pharmacologically inhibit the Hippo pathway. As expected, XMU-MP-1 attenuated the antiproliferative properties of NLOC-015A in both H1975 and H1299 cells, while veterporfin, an activator of the Hippo pathway, produced a reverse effect (**Figures 13D**).

### NL0C-015A Compromised Colony-Formation, Invasion and Spheroid Forming Abilities of NSCLC

**Figure 14:**
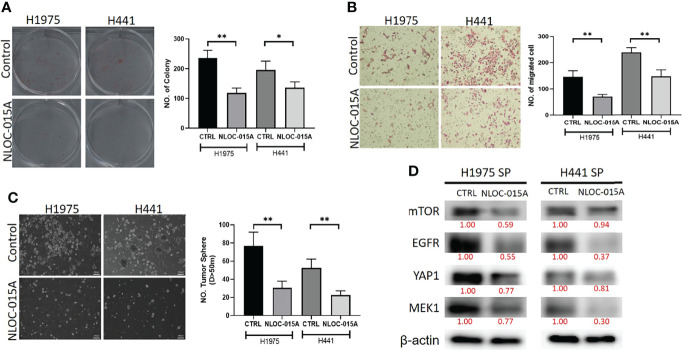
NL0C-015A compromised the oncogenic phenotypes of non-small-cell lung cancer (NSCLC). Graphical representation of the inhibitory effects of NL0C-015A on the **(A)** colony-forming, **(B)** migratory abilities, and **(C)** spheroid forming of H441 and H1975 cells. **(D)** Western blot analysis showing that NL0C-015A suppressed expression levels of *EGFR, mTOR, YAP1,* and *MEK1* in H441 and H1975 cells compared to their vehicle-treated counterparts. *p < 0.05, **p < 0.01.

We evaluated the effects of NL0C-015A on the oncogenic phenotypes of NSCLC cells. Our results revealed that treatments with NL0C-015A significantly inhibited the colony-formation (**Figure 14A**), migratory abilities (**Figure 14B**), and spheroid forming abilities (**Figure 14C**) of H441 and H1975 cells. Western blot analysis revealed that the spheroid-forming inhibition effect of NLOC-015A was concomitantly associated with decreased expression levels of EGFR, MEK1, mTOR and YAP1 (**Figure 14D**) in both the H1975 and H441 cell lines. Collectively, our results demonstrated that NL0C-015A compromised the oncogenic phenotypes of NSCLC cells *via* modulation of the YAP1, EGFR, MEK1, and mTOR signaling pathways.

## Discussion

Lung cancer is one of the most prevalent malignancies with a devastating prognosis all over the world (1). The resistance to several generations of EGFR-TKIs has necessitated the search for new drugs with multitarget potential that offer less chance of developing drug resistance. Results of the present study implied the roles of EGFR/MAP2K1/mTOR/YAP1 signaling pathways in the progression, therapeutic resistance, immune-invasive phenotypes, and worse prognoses of NSCLC. In addition, we documented the therapeutic efficacy of NLOC-015A, a multitarget small-molecule inhibitor of EGFR/MAP2K1/mTOR/YAP1 against NSCLC.

Our analysis of TCGA cohorts of NSCLC revealed that dysregulation and genetic alterations of the EGFR, MAP2K1, mTOR, and YAP1 signaling pathways were associated with the progression, therapeutic response, and worse prognosis of NSCLC. In line with our findings, EGFR/MAP2K1/mTOR/YAP1 were implicated in aggressive phenotypes of NSCLC (62). Despite the implicative role of EGFR in initiation and progression, experimental studies have revealed a potent downregulation of EGFR through metastatic progression (63, 64). This progressive decrease expression of EGFR during oncogenic metastasis is further supported herein by our data utilizing the TCGA dataset where EGFR expression is significantly downregulated in metastatic lung cancer compare to the primary tumor. Our results are also in consistent with clinical data that reported the downregulation of EGFR2 in metastatic tumor when compared to the primary tumors (65). Activation of YAP as manifested by gene fusion, amplification of genomic locus, increased expression, or enhanced nuclear translocation is a commonly observed features of malignant tumors (66–70), suggesting that YAP activation contributes to tumor progression and metastasis (66). Indeed, YAP overexpression in non-transformed epithelial cells results in epithelial-to-mesenchymal transition (EMT), a critical process for cancer metastasis (69, 71). Several experimental studies have implicated YAP, TAZ, or TEADs in metastasis of numerous cancers including melanoma, lung, breast, gastric, ovarian, oral squamous cell carcinoma, and colorectal cancers (72–74). However, these experimental data were supported by human patient data only in pancreatic cancer (75), breast cancer (76), and prostate cancer (77); where YAP or TAZ expression and nuclear localization is increased in metastatic tumors when compared to primary tumors. Conversely, and in line with the results of the present study, some studies found that YAP expression is inversely correlated with metastasis (78–80). Clinical studies using lung tumor specimen also revealed the association of low YAP expression with lung tumor metastasis (80). YAP expression also inversely correlated lymph node and distant metastasis in breast cancer (78, 79). However, it is reported that YAP activities is mainly dependent on concerted activities of its binding partner, TEAD (71), which we found to be significantly overexpressed in metastatic lung cancer compare to the primary tumor. This opposing expression levels of YAP and TEAD in primary and metastatic lung cancer warrant further experimental study. Altogether these data suggested that YAP mediated tumor metastasis in cancer context dependent manner. However, our findings therefore suggest the pro-apoptotic switch in the role of EGFR and YAP1 during lung cancer metastasis. However, further experimental studies are required to fully elucidate the role of YAP/EGFR in lung cancer metastasis.

Hippo pathway is a tumor suppressor pathway that is mainly regulated by a phosphorylation-dependent protein kinase cascade (81). MST1/2 kinase phosphorylates LATS1/2, which in turn phosphorylates and inhibits YAP/TAZ, *via* cytoplasmic sequestration, ubiquitination and proteasomal degradation (81, 82). Therefore, the decrease expression of p-YAP in primary tumor (Grade 1) when compared with normal tissue and further decease phosphorylation from grade II to III indicated the inhibition of Hippo pathway, and support it oncogenic contribution to the advance stage of NSCLC. Decreases phosphorylation and increase YAP activities have been implicated in resistance to a number of cancer drugs (20, 21) including the first- and second-generation EGFR-TKIs (22), and treatment of lung cancer with growth factor (vascular endothelial growth factor (VEGF) and YAP) inhibitors provided promising results in previous studies (3, 23). Contrary to our observations, studies have reported increase phosphorylation of mTOR in advance tumor stages. Rausch et al. (83) reported that the high level of p-mTOR was significantly correlated with advanced T-stage of renal carcinoma (83), while Zhang et al. (84), reported that the expressions of p-mTOR in NSCLC increases from stage I (21.1%), stage II (32.4%), to stage III-IV (56.3%) (84). It appears that bulk RNA sequencing data does not support the oncogenic role of p-mTOR in advance stage of NSCLC.

Tumor microenvironment (TME) is a complex system comprising of various cells including the stroma cells, tumor cells, chemokines, cytokines, microvessels and infiltrating immune cells which make up the largest portion of the TME (85). Tumor initiation depends on vital contributions from the tumor microenvironment (TME) and host immune alterations (86). Hence, we evaluated the potential role of EGFR, MAP2K1, mTOR, and YAP1 in promoting the infiltration of tumor immune cells within the TME (25). Interestingly, our results suggested that high expression levels of the gene signature would negatively regulate infiltration levels of cytotoxic lymphocytes, Th cells, and common lymphoid progenitors while inducing increased tumor infiltration levels of monocytes, granulocyte-monocyte progenitors, and immunosuppressive cells that are known mediators of T-cell exclusion.

We further explored the single-cell RNA sequencing (scRNA-seq) and found that Patients with primary NSCLC exhibited higher abundancy of the immune cells within the TME when compared with the malignant cells and stromal cells. Specifically, we found that the TME is mostly populated by monocytes and macrophages, regulatory T cells and Exhausted CD8 T Cells while a very low level of active CD8 T cells was found within the TME of patients with primary NSCLC. Similarly, our single cell RNA sequencing data demonstrated that MAP2K1, mTOR, YAP1 and EGFR were predominantly located on monocytes/macrophages, Treg and exh. CD8 T cell within the TME of patients with primary NSCLC which further implied MAP2K1, YAP1, EGFR and mTOR’s role in remodeling the immune microenvironment. we further investigated the expression of the genes in different states of macrophages, and the results demonstrated that genes’s expression were significantly higher in M2 macrophages compared to M0 and M1 macrophages, suggesting that EGFR/MTOR/YAP1/MAP2K1 are involved in M2 tumor associated macrophage polarization, which was an essential step for the remodeling of tumor immune microenvironment (TIME) (55). These above findings suggest that EGFR/MTOR/YAP1/MAP2K1 activation in combination with the polarization of M2 macrophages and infiltration of regulatory T cells and exhausted T cells led to remodeling of TIME and marked tumor progression. Collectively, our analysis of single cell sequencing data suggested that the TME of primary tumor of NSCLC are enormously immunosuppressive than the metastatic tumor and are populated by high expression levels of EGFR/MAP2K1/MTOR/TEAD1/YAP1 within tumor microenvironment of NSCLC. Our study has increase our understanding of the complex regulatory mechanism of the interaction within the TME of NSCLC.

The impaired function of tumor-infiltrating cytotoxic immune cells and the shift towards immunosuppressive cells may induce immunosuppressive phenotypes within the TME and may critically affect the body’s antitumor immune response, favoring tumor growth, invasiveness, drug resistance, and metastasis (87, 88). Indeed, we found that the immune infiltration-mediating roles of EGFR/MAP2K1/mTOR/TEAD1/YAP1 led to overall decreased levels of active cytotoxic lymphocytes in NSCLC tumors and achieved shorter survival durations of cohorts with dysfunctional T-cell phenotypes. Collectively our results suggested that EGFR, MAP2K1, mTOR, TEAD1, and YAP1 could mediate invasive tumor phenotypes and worsen prognoses *via* mechanisms involving both T-cell exclusion and dysfunctional phenotypes. Therefore, targeting inhibitors of these oncogenes appears to be an effective strategy for treating and managing NSCLC. Drug sensitivity analysis shows that in contrast to the association of YAP1/EGFR/MAP2K1 with drug resistance, high expressions of mTOR are favorable to cancer chemotherapy. Hence, mTOR hold promise in cancer therapy. Indeed, many mTOR inhibitors have been approved to treat human cancer (89), while more mTOR inhibitors are being evaluated in clinical trials (90). In addition, combining mTOR inhibitor with target therapy and standard chemotherapeutic drugs have yielded superior clinical outcome than individual therapy in several cancer types (91–94). Altogether, our findings suggested that YAP1/EGFR/MAP2K1/MTOR are potential novel markers for drug screening and therapy exploration

Analysis of receptor-ligand interactions *via* molecular docking is a widely used approach for simulating small-molecule interactions with a protein target and for predicting biological activities of compounds (95). Non-covalent interactions, including hydrogen bonds, hydrophobic contacts, and ionic interactions, play pivotal roles in stabilizing interactions of small molecules with protein targets (96). Subsequently, we explored the potential of this compound as a therapeutic target against YAP1/EGFR/MEK1/mTOR through an *in silico* ligand-receptor interaction study. Our molecular docking analysis revealed that NLOC-015A bound to YAP1, EGFR, MEK1 and mTOR with strong binding efficacies ranging –8.4 to –9.50 kcal/mol. NLOC-015A bound to the targets by several conventional H-bonds, halogen bonds, and alkyl and multiple π-interactions. These non-covalent interactions play pivotal roles in stabilizing small molecules within the binding domain of a target (96). In addition, several van der Waals forces and hydrophobic contacts were found around the NLOC-015A backbone with respective amino acid residues of target-binding pockets. These van der Waal forces are non-covalent interactions and would create a strong cohesive environment, that could stabilize the complex (97). Interestingly, compared to osimertinib’s binding affinity, NLOC-015 bound with higher efficacy to TK domains of T790M- and T790M/C797S-mutant bearing EGFR. These results hinted at the potential inhibitory roles of NLOC-015A on the expressions and activities of YAP1/EGFR/MEK1/mTOR, and consequently its therapeutic potential for treating NSCLC.

ADMET PKs are drug-like properties that determine the fate of a therapeutic agent and have been criteria for judging the success or failure of many clinical drugs. Therefore, analysis of these parameters has become relevant in drug design and development pipelines (98). Fortunately, our results revealed adherence to Lipinski’s rules, and good ADMET and drug-like properties of NLOC-015A. P-Glycoprotein (P-gp) is a multidrug-resistance protein 1 (MDR1) which pumps drugs and various compounds out of cells (99). NLOC-015A is a not a substrate for P-gp. These results further suggested that NLOC-015A would have good absorption, permeability, and retention and would achieve optimum drug delivery and a high therapeutic index. In addition, the BBB permeation ability of NLOC-015A is an added advantage which suggests its potential for crossing the BBB and offering a treatment window against lung-brain-metastasized NSCLC. Summing up, NLOC-015A possesses good translational relevance as a potential anti-NSCLC drug candidate worthy of experimental validation.

Interestingly, we provide preclinical evidence of the anti-NSCLC properties of NLOC-015A of suppressing the proliferation of NSCLC in *in vitro* model. We demonstrated that NLOC-015A inhibited the proliferation 9 cell line panel of NSCLC with concomitant downregulation of expression levels of oncogenic molecules, including EGFR, mTOR, Akt, NF-κB, YAP1, MEK signaling network. Furthermore, we demonstrated that NLOC-015A suppressed the oncogenic attributes (colony formation, migration and sphere formation) of H1975 and H441 cells with concomitant downregulation of expression levels of oncogenic molecules. Thus, the inhibitory effect of NLOC-015A on these onco-immunogenic signatures could be of translational relevance for immunotherapy.

## Conclusions

In conclusion, our analysis of clinical NSCLC cohorts revealed that dysregulation of the EGFR/MAP2K1/mTOR/YAP1 signaling pathways was associated with the progression, therapeutic resistance, immune-invasive phenotypes, and worse prognoses of NSCLC. NLOC-015A, a novel multi-target small molecule, has ADMET PK properties of a good drug-like candidate and efficiently suppressed the proliferation and oncogenic phenotypes of NSCLC with concomitant inhibition of EGFR, mTOR, Akt, NF-κB, YAP1, MEK signaling network. We, therefore, suggest that NLOC-015A might represent a new candidate for treating NSCLC *via* acting as a multi-target inhibitor of EGFR, mTOR, YAP1, and MEK signaling network in NSCLC.

## Data Availability Statement

The datasets presented in this study can be found in online repositories. The names of the repository/repositories and accession number(s) can be found below: NCBI with BioSample SAMN26207491’ and SAMN26207492.

## Author Contributions

BL carried out the study and wrote the manuscript; H-SH synthesized and provided the drugs; AW and H-SH designed and oversaw the study. All authors have read and agreed to the published version of the manuscript.

## Funding

H-SH was funded by the Ministry of Science and Technology, Taiwan (MOST110-2314-B-038-120).

## Conflict of Interest

The authors declare that the research was conducted in the absence of any commercial or financial relationships that could be construed as a potential conflict of interest.

## Publisher’s Note

All claims expressed in this article are solely those of the authors and do not necessarily represent those of their affiliated organizations, or those of the publisher, the editors and the reviewers. Any product that may be evaluated in this article, or claim that may be made by its manufacturer, is not guaranteed or endorsed by the publisher.
