# Supplementary material for: Leveraging Bulk and Single-Cell RNA Sequencing Data of NSCLC Tumor Microenvironment and Therapeutic Potential of NLOC-15A, A Novel Multi-Target Small Molecule
Source: Front Immunol. 2022 May 17;13:872470. doi: 10.3389/fimmu.2022.872470 (PMC9152008; doi:10.3389/fimmu.2022.872470)
Supplement: Supplementary file 1 [file DataSheet_1.docx]

**
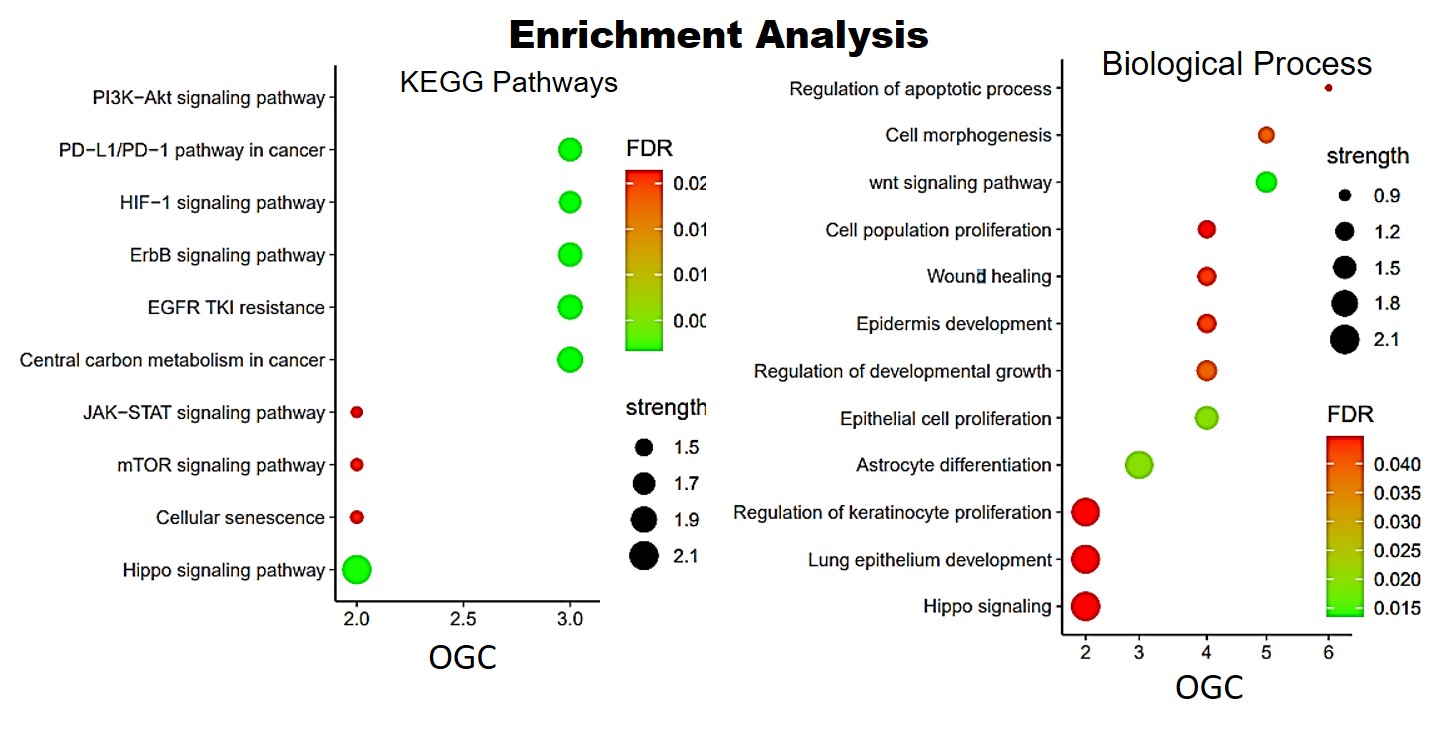
Figure S1:** Enrichment analysis of *EGFR/MAP2K1/mTOR/TEAD1/YAP1* mediated abnormal gene expression in lung cancer


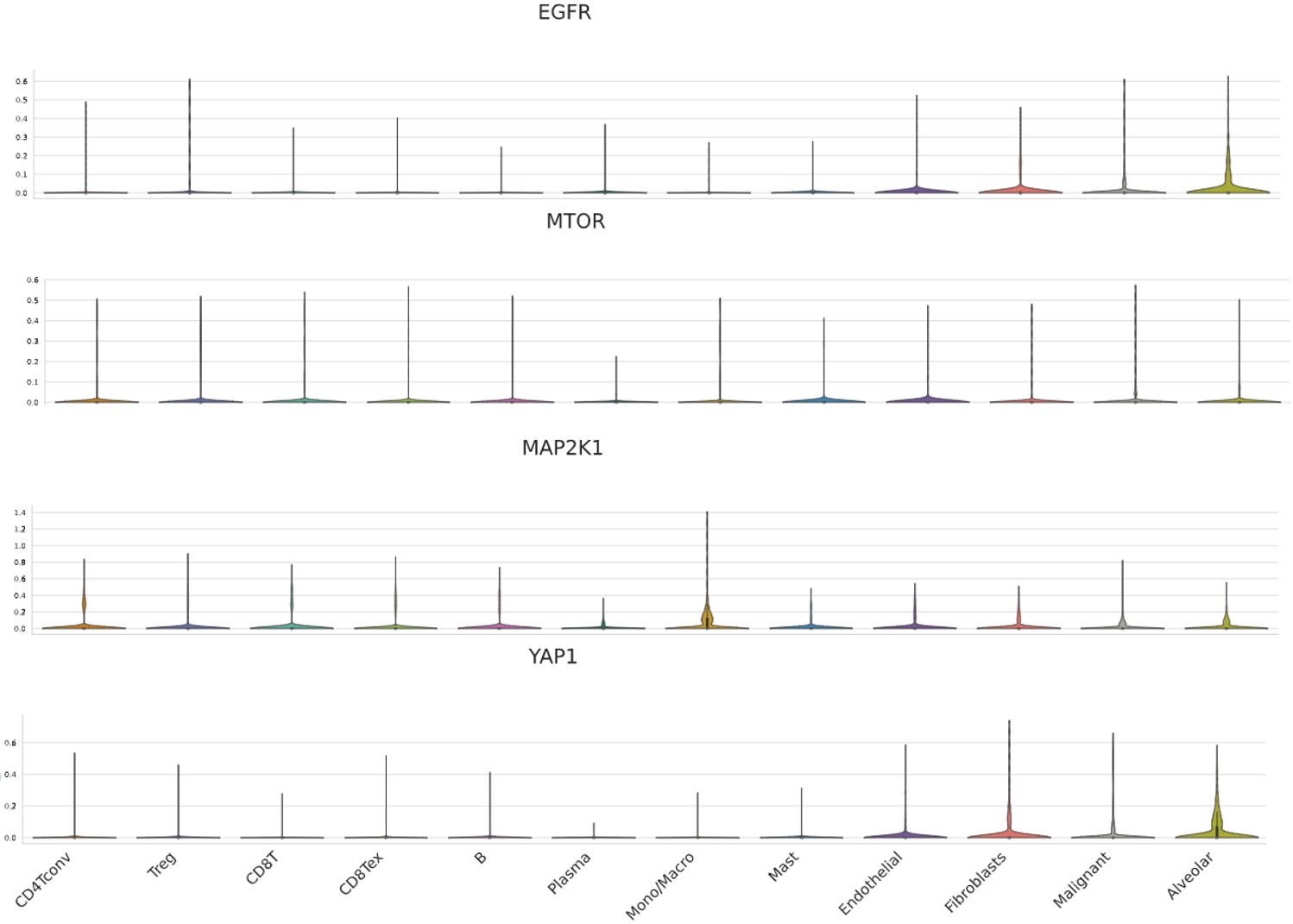


**Figure S2:** Figure S2: *EGFR/MAP2K1/mTOR/YAP1* expression level in different cell type based on the single-cell RNA sequencing dataset of NSCLC


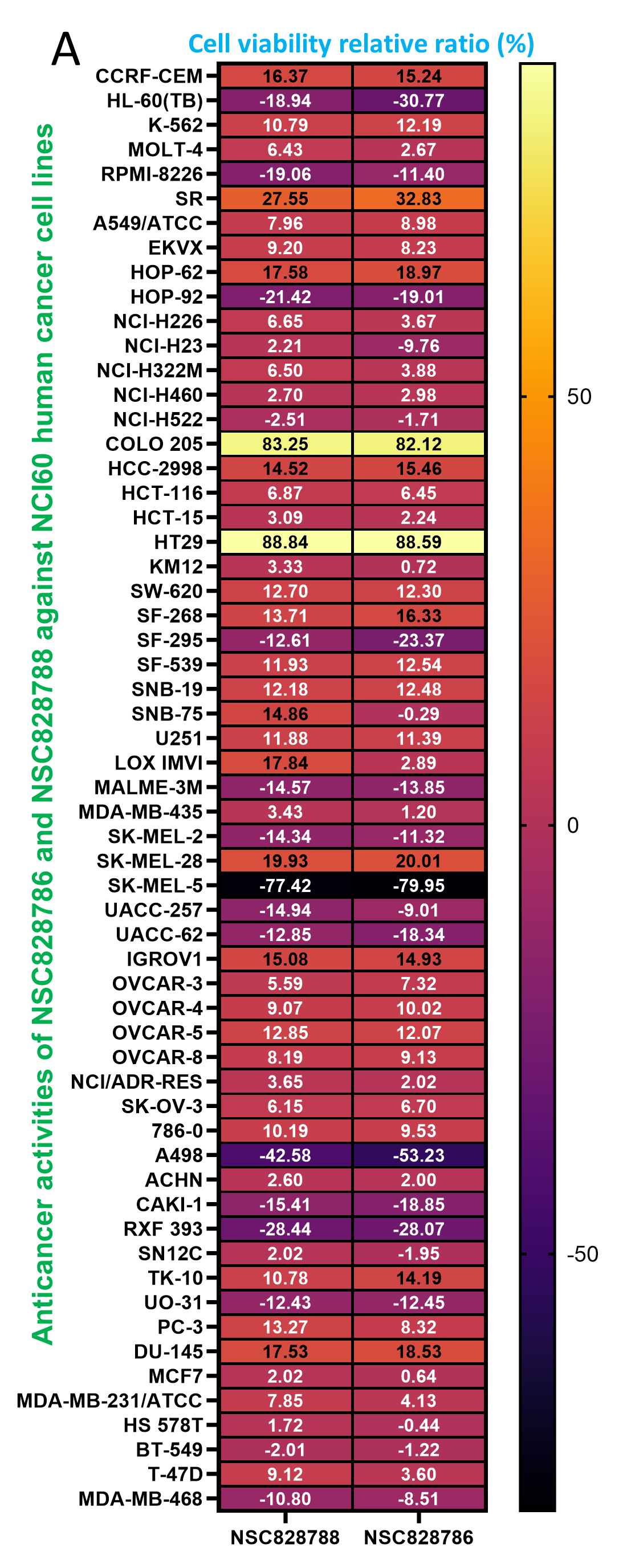


**Figure S3**: Heatmap of *in vitro* anticancer activities of NSC828786 and NSC828788 against NCI-60 human tumor cancer cell lines.
